# Molecular Epidemiology of *Acinetobacter calcoaceticus*-*Acinetobacter baumannii* Complex Isolated From Children at the Hospital Infantil de México Federico Gómez

**DOI:** 10.3389/fmicb.2020.576673

**Published:** 2020-10-15

**Authors:** Jetsi Mancilla-Rojano, Sara A. Ochoa, Juan Pablo Reyes-Grajeda, Víctor Flores, Oscar Medina-Contreras, Karina Espinosa-Mazariego, Israel Parra-Ortega, Daniela De La Rosa-Zamboni, María del Carmen Castellanos-Cruz, José Arellano-Galindo, Miguel A. Cevallos, Rigoberto Hernández-Castro, Juan Xicohtencatl-Cortes, Ariadnna Cruz-Córdova

**Affiliations:** ^1^ Laboratorio de Investigación en Bacteriología Intestinal, Subdirección de Gestión de la Investigación, Hospital Infantil de México Federico Gómez, CDMX, Mexico; ^2^ Facultad de Medicina, Posgrado en Ciencias Biológicas, Universidad Nacional Autónoma de México, CDMX, Mexico; ^3^ Subdirección de Desarrollo de Aplicaciones Clínicas, Instituto Nacional de Medicina Genómica, Ciudad de México, Mexico; ^4^ Unidad de Genómica Avanzada, Laboratorio Nacional de Genómica para la Biodiversidad, Irapuato, Mexico; ^5^ Unidad de Investigación Epidemiológica en Endocrinología y Nutrición, Hospital Infantil de México Federico Gómez, CDMX, Mexico; ^6^ Departamento de Laboratorio Clínico, Laboratorio Central, Hospital Infantil de México Federico Gómez, CDMX, Mexico; ^7^ Departamento de Epidemiología Hospitalaria, Hospital Infantil de México Federico Gómez, CDMX, Mexico; ^8^ Unidad de investigación en Enfermedades Infecciosas, Subdirección de Gestión de la Investigación, Hospital Infantil de México Federico Gómez, CDMX, Mexico; ^9^ Centro de Ciencias Genómicas, Programa de Genómica Evolutiva, Universidad Nacional Autónoma de México, Cuernavaca, Mexico; ^10^ Departamento de Ecología de Agentes Patógenos, Hospital General Dr. Manuel Gea González, CDMX, Mexico

**Keywords:** *Acinetobacter baumannii*, *Acinetobacter calcoaceticus*-*Acinetobacter baumannii* complex, intensive care unit, resistance, molecular typing

## Abstract

The *Acinetobacter calcoaceticus*-*baumannii* (*Acb*) complex is regarded as a group of phenotypically indistinguishable opportunistic pathogens responsible for mainly causing hospital-acquired pneumonia and bacteremia. The aim of this study was to determine the frequency of isolation of the species that constitute the *Acb* complex, as well as their susceptibility to antibiotics, and their distribution at the Hospital Infantil de Mexico Federico Gomez (HIMFG). A total of 88 strains previously identified by Vitek 2®, 40 as *Acinetobacter baumannii* and 48 as *Acb* complex were isolated from 52 children from 07, January 2015 to 28, September 2017. *A. baumannii* accounted for 89.77% (79/88) of the strains; *Acinetobacter pittii*, 6.82% (6/88); and *Acinetobacter nosocomialis*, 3.40% (3/88). Most strains were recovered mainly from patients in the intensive care unit (ICU) and emergency wards. Blood cultures (BC) provided 44.32% (39/88) of strains. The 13.63% (12/88) of strains were associated with primary bacteremia, 3.4% (3/88) with secondary bacteremia, and 2.3% (2/88) with pneumonia. In addition, 44.32% (39/88) were multidrug-resistant (MDR) strains and, 11.36% (10/88) were extensively drug-resistant (XDR). All strains amplified the *bla*_OXA-51_ gene; 51.13% (45/88), the *bla*_OXA-23_ gene; 4.54% (4/88), the *bla*_OXA-24_ gene; and 2.27% (2/88), the *bla*_OXA-58_ gene. Plasmid profiles showed that the strains had 1–6 plasmids. The strains were distributed in 52 pulsotypes, and 24 showed identical restriction patterns, with a correlation coefficient of 1.0. Notably, some strains with the same pulsotype were isolated from different patients, wards, or years, suggesting the persistence of more than one clone. Twenty-seven sequence types (STs) were determined for the strains based on a Pasteur multilocus sequence typing (MLST) scheme using massive sequencing; the most prevalent was ST 156 (27.27%, 24/88). The Clustered Regularly Interspaced Short Palindromic Repeats (CRISPR)-Cas I-Fb system provided amplification in *A. baumannii* and *A. pittii* strains (22.73%, 20/88). This study identified an increased number of MDR strains and the relationship among strains through molecular typing. The data suggest that more than one strain could be causing an infection in some patient. The implementation of molecular epidemiology allowed the characterization of a set of strains and identification of different attributes associated with its distribution in a specific environment.

## Introduction

The *Acinetobacter* genus includes species widely disseminated in nature, mostly in water and soil; some of these species are considered opportunistic pathogens that are relevant for their association with health-care associated infections (HAIs). The clinically important species in this genus are *Acinetobacter pittii*, *Acinetobacter nosocomialis*, and *Acinetobacter baumannii*, with the last being the most important epidemiologically and frequently isolated from the intensive care unit (ICU), causing infections such as ventilator-associated pneumonia, bacteremia, urinary tract infection, meningitis, and wound infection (Dexter et al., 2015; Wong et al., 2017).

The *Acinetobacter calcoaceticus*-*baumannii* complex (*Acb*) harbored six species: *A. calcoaceticus*, *A. baumannii*, *A. pittii*, *A. nosocomialis*, *Acinetobacter seifertii*, and *Acinetobacter dijkshoorniae* (Gerner-Smidt et al., 1991; Nemec et al., 2011, 2015). The *Acb* complex cause hospital-acquired pneumonia and bacteremia in critically ill or immunocompromised patients (Wong et al., 2017; Vazquez and Kollef, 2018). The species of the complex are phenotypically indistinguishable and molecular methods are required for its correct identification (Gerner-Smidt et al., 1991). The participation of *A. calcoaceticus* in clinical infections remains unclear compared with other species, in which clinical importance has been demonstrated (Peleg et al., 2008; Nemec et al., 2015; Cosgaya et al., 2016). *A. baumannii* is one of the most difficult bacteria to contain in a hospital environment. Additionally, it is included in the list of priority pathogens resistant to antibiotics, owing to the resistance it has acquired to different antibiotics such as carbapenems and cephalosporins limiting the therapeutic options for the treatment of infections caused by this pathogen (Higgins et al., 2010; Shrivastava et al., 2018).

The pulsed-field gel electrophoresis (PFGE), multilocus sequence typing (MLST), and amplified fragment length polymorphism (AFLP) methods have been used to type clinical strains of *A. baumanni* (Limansky et al., 2004; Rafei et al., 2014). In hospitals in Spain and Germany, a total of 20 allelic profiles or sequence types (STs) were identified through MLST, and these results agreed with those generated by PFGE, suggesting MLST as a tool for the molecular epidemiological study of clinical strains of *A. baumannii* (Bartual et al., 2005). However, there are other studies where according to the tool used, the typing of the strains differs completely; the relationships between clinical isolates that were determined through PFGE revealed that isolates were not closely related (Evans et al., 2008). Through nucleotide analysis of the *bla*_OXA-51_ gene sequence, two closely related groups were identified. The sequencing of the *bla*_OXA-51_ gene, being only one genetic element, yielded a less-defined clonal relationship than PFGE analyses, where the whole genome is digested and analyzed to establish whether there is a clonal relationship between the strains under study (Evans et al., 2008).

The Clustered Regularly Interspaced Short Palindromic Repeats (CRISPR)-Cas system has been proposed for typing bacterial strains. Two CRISPR-Cas systems have been identified in strains of *A. baumannii* from military and children’s hospitals. An analysis of the nucleotide sequences of the CRISPR-Cas systems (CRISPR-AYE and *Acinetobacter baylyi* ADP systems) grouped the isolates into two clonal complexes and provided information about the evolution of these complexes (Hauck et al., 2012). At the same time, the CRISPR-Cas I-Fb system has been proposed for the subtyping of strains (Karah et al., 2015).

Genome sequencing is used to characterize and establish genetic relationships among isolates. Clinical strains identified as *Acb* complex were typed using PFGE, MLST, and single nucleotide polymorphism (SNP) analyses. SNP analysis was more discriminatory than those obtained by PFGE and MLST for the identification of clones and their association with outbreaks (Fitzpatrick et al., 2016).

*A. baumannii* mortality is approximately 14.5% and it has been associated with HAIs in tertiary level hospitals in Mexico (Bocanegra-Ibarias et al., 2015). Furthermore, the Mexican strains that have been characterized are distinguished by their resistance to imipenem and meropenem, as well as being associated with the amplification of carbapenemases such as OXA-23, OXA-239, and OXA-58. An epidemiological aspect of relevance is the different STs distributed among Mexican hospitals, belonging to clonal complexes 636 and 92, presenting ST208, ST369, and ST758 (Alcántar-Curiel et al., 2014; Tamayo-Legorreta et al., 2014; Gonzalez-Villoria et al., 2016). However, information about other species related to the *Acb* complex has not been available. The *Acb* complex poses a major challenge to this genus, suggesting a very recent diversification of those species, and events of homologous recombination can probably be contributed to a homogenous gene composition (Mateo-Estrada et al., 2019).

The aim of this study was to determine the frequency of isolation of the species that constitute the *Acb* complex, as well as the susceptibility to antibiotics, and their distribution at the Hospital Infantil de Mexico Federico Gomez (HIMFG). To achieve this aim, the *Acinetobacter* species were distinguished when the strains were identified as the *Acb* complex using a collection of 88 strains from 52 children from 07, January 2015 to 28, September 2017, previously identified as the *A. baumannii* and *Acb* complex. Then, after the susceptibility profile was determined, four of the most frequent *bla_OXA_* genes were detected, the plasmid profile, pulsotype, and sequence type were established and the CRISPR-Cas system was carried out for all strains.

## Materials and Methods

### Identification by MALDI-TOF Biotyper

In this study, all strains among 07, January 2015 to 28, September 2017 were considered and identified as the *A. baumannii* or *Acb* complex, from patients with or without HAIs. If one patient had more than one strain, all strains were included in this study. The strains were previously identified at the Central Clinical Laboratory at the HIMFG using the Vitek® 2 automated system (BioMérieux, Marcy l’Étoile France), and they were subsequently reidentified by matrix-assisted laser desorption/ionization time-of-flight (MALDI-TOF) Biotyper (mass spectrometer, Bremen, Germany). Each strain was spread onto Brucella blood agar (BD Difco, Madrid, Spain), and one colony was placed on a metallic card for analysis (Bruker Daltonics Ultraflextreme, Bremen, Germany). The sample allowed to dry at room temperature; 1 μl of formic acid (70%) was placed on each well to dry at room temperature, and 1 μl of matrix [saturated solution of α-cyano-4-hydroxycinnamic acid (HCCA; Bruker Daltonics Ultraflextreme, Bremen, Germany) in 50% acetonitrile (Sigma, California, United States) and 2.5% trifluoroacetic acid were subsequently added (Sigma, California, United States)]. The spectra were analyzed using the MALDI Biotyper software Bruker Daltonics Ultraflextreme 3.1 (Bremen, Germany) and were compared with a database using identification criteria at the species level with a score between 1.7 and 1.9.

### Antibiotic Susceptibility Test

Antibiotic susceptibility testing was performed using a Vitek® 2 automated system (BioMériux, Marcy l’Étoile, France). The antibiotics considered included penicillins (piperacillin); β-lactam combination agents (ampicillin-sulbactam and piperacillin-tazobactam); cephems (cefepime and ceftriaxone); carbapenems (imipenem); a lipopeptide (colistin); aminoglycosides (gentamicin); fluoroquinolones (ciprofloxacin); and folate pathway antagonists (trimethoprim-sulfamethoxazole), according to the Clinical and Laboratory Standards Institute (CLSI, 2018). Colistin susceptibility by the broth microdilution method according to CLSI was determined. *Escherichia coli* ATCC®25922 and *Pseudomonas aeruginosa* ATCC®27853 were used as quality controls; including *A. baumannii* ATCC®19606 as an internal control. Susceptibility to tigecycline was interpreted according to the United States Food and Drug Administration (FDA) breakpoints for *Enterobacteriaceae*. The multidrug-resistant (MDR) profile was defined as the strains resistant to three or more antimicrobial classes, and the extensively drug-resistant (XDR) profile was defined as the strains nonsusceptible to ≥1 agent in all but ≤2 categories (Magiorakos et al., 2012).

### Amplification of *bla*_OXA-LIKE_ Genes

Genomic DNA of strains was obtained with the Quick-DNA Universal kit (Zymo, Irvine, California, United States). *bla*_OXA-LIKE_ genes were amplified by PCR using the specific primers listed in Table 1. PCR assays were performed using the following thermocycling conditions: 94°C for 5 min; 30 cycles at 94°C for 25 s, 52°C for 40 s, and 72°C for 50 s; and a final step at 72°C for 6 min. *A. baumannii* ATCC®19606 was used as a positive control for *bla*_OXA-51_.

**Table 1:** Primers used to amplify *bla_OXA-LIKE_* genes.

Gene	Sequence 5' – 3'	Amplified size (bp)	Reference
*bla_OXA-23_*	F: GATCGGATTGGAGAA CAGA R: ATTTCTGACCGCATTTCCAT	501	Hujer et al. (2006)
*bla_OXA-24_*	F: GGTTAGTTGGCCCCCTTAAA R: AGTTGACGCAAAAGGGGATT	246	Hujer et al. (2006)
*bla_OXA-51_*	F: TAATGCTTTGATCGGCCTTG R: TGCATTGCACTTCATCTTGG	353	Hujer et al. (2006)
*bla_OXA-58_*	F: AAGTATTGGGGCTTGTGCTG R: CCCCTCTGCGCTCTACATAC	599	Hujer et al. (2006)

OXA-23 (*bla_OXA-23_*), OXA-24 (*bla_OXA-24_*), OXA-51 (*bla_OXA-51_*), and OXA-58 (*bla_OXA-58_*).

### Plasmid DNA Profiles

The extraction of plasmid DNA was performed using the technique of Eckhardt (1978). A colony of each strain grown first on Brucella blood agar was cultured in 3 ml of Luria-Bertani (LB) broth (BD Difco, Madrid, Spain) with constant stirring (200 rpm) at 37°C for 15 h. Subsequently, 100 μl of this bacterial culture was incubated in 5 ml of LB broth under agitation at 37°C for 2.5 h. Finally, 1 ml bacterial culture was taken and centrifuged for 8 min at 14,000 rpm. The pellet was dissolved in 500 μl of cold sterile water, mixed with 1 ml of 0.3% sarcosyl solution, and then centrifuged at 14,000 rpm for 6 min. The pellet was incubated with 40 μl of 20% Ficoll in 10:1 TE buffer, kept on ice for 15 min and mixed with 20 μl of lysis solution [0.4 mg/ml RNase, 1 mg/ml bromophenol blue, 80 μl lysozyme (20 mg/ml in water)]. After 30 μl of SDS (10%) was added to each well, the samples were run on a 0.75% agarose gel (Promega, Wisconsin, United States) under the following conditions: 100 V for 15 min in 1X TBE buffer (AMRESCO, United States) without completely covering the gel and with the negative polarity inverted. After this time, 1X TBE was added to the chamber in a cold room until the gel was covered, and the samples were placed. The electrophoretic shift was performed with the poles in the standard orientation at 40 V for 90 min and thereafter at 100 V for 21 h. The gel was stained with ethidium bromide for visualization.

### PFGE Assay

The 88 strains were seeded and incubated at 37°C for 18 h. PFGE assay was performed for all strains as described by Mancilla-Rojano et al. (2019). Briefly, colonies cultured on Brucella blood agar were selected and suspended in 1 ml of negative Gram suspension buffer (100 mM Tris-HCl and 100 mM EDTA 100 pH 8). The bacterial suspension was embedded into 1% agarose plugs (SeaKem, Cambrex, Rockland, United States) and lysed with 5 ml of lysis buffer at pH 8.0 [0.5 M Tris-HCl, 0.5 M EDTA, 1% N-lauryl sarcosine sodium salt, and 25 μl of proteinase K (20 mg/ml)]. Afterward, the samples were digested with the ApaI enzyme (Promega, Wisconsin, United States), and the chromosomal DNA obtained was subjected to electrophoresis on 1% agarose gels (Bio-Rad, Hercules, California, United States) using in the CHEF MAPPER system (Bio-Rad, Hercules, California, United States) using 0.5X TBE (AMRESCO, United States) under the following conditions: initial time 5.0 s, final time 30.0 s, 6 V/cm, inclination angle 120, and running time 24 h. The lambda marker (Biolabs, Hertfordshire, England, United Kingdom) was used as a molecular weight marker. The electrophoresis gels were stained with 0.5 mg/ml ethidium bromide for 40 min and visualized under UV light. The DNA fragment patterns generated by PFGE were analyzed and compared using NTSYS software version 2.2 (Applied Biostatistics, Setauket, New York, United States) with the unweighted pair group method using the arithmetic average (UPGMA) algorithm and the DICE correlation coefficient. The relatedness degree was assessed according to the criteria established by Tenover et al. (1995).

### MLST Assay

Amplification of the fragments was carried out according to the MLST protocol with some modifications (Diancourt et al., 2010). The primers to amplify the *fusA* (elongation factor EF-G) gene were designed using the genome of *A. baumannii* AYE (GCA_000069245.1). To perform massive sequencing, the following adaptors were incorporated in each primer: F: 5'- TCG TCG GCA GCG TCA GAT GTG TAT AAG AGA CAG-3' and R: 5'- GTC TCG TGG GCT CGG AGA TGT GTA TAA GAG ACA-3' (Table 2).

**Table 2:** Primers used to amplify genes to determine STs by multilocus sequence typing (MLST).

Gene	Sequence 5'-3'	Amplified size (bp)	
*cpn60*	F: TCG TCG GCA GCG TCA GAT GTG TAT AAG AGA CAG ACT GTA CTT GCT CAA GC R: GTC TCG TGG GCT CGG AGA TGT GTA TAA GAG ACA GTT CAG CGA TGA TAA GAA GTG G	480	Diancourt et al. (2010)
*fusA*	F: TCG TCG GCA GCG TCA GAT GTG TAT AAG AGA CAG ACA ATT ACC TCT GCT GCA ACA R: GTC TCG TGG GCT CGG AGA TGT GTA TAA GAG ACA TCA TCG CAG GTT TAC GTG CT	633	This study
*gltA*	F: TCG TCG GCA GCG TCA GAT GTG TAT AAG AGA CAG AGA TGT ATT GGC CTC AGG TCA CTT R: GTC TCG TGG GCT CGG AGA TGT GTA TAA GAG ACG GGT TTA CTT TGT AGT CAC GGT CTG	545	Diancourt et al. (2010)
*pyrG*	F: TCG TCG GCA GCG TCA GTA GTG TAT AAG AGA CAG GGT GTT GTT TCA TCA CTA GGW AAA GG R: GTC TCG TGG GCT CGG AGA TGT GTA TAA GAG ACA GAT AAA TGG TAA AGA YTC GAT RTC ACC	434	Diancourt et al. (2010)
*recA*	F: TCG TCG GCA GCG TCA GAT GTG TAT AAG AGA CAG CCT GAA TCT TCY GGT AAA AC R: GTC TCG TGG GCT CGG AGA TGT GTA TAA GAG ACA GGT TTC TGG GCT GCC AAA CAT TAC	425	Diancourt et al. (2010)
*rplB*	F: TCG TCG GCA GCG TCA GAT GTG TAT AAG AGA CAG GTA GAG CGT ATT GAA TAC GAT CCT AAC C R: GTC TCG TGG GCT CGG AGA TGT GTA TAA GAG ACA GCA CCA CCA CCR TGY GGG TGA TC	472	Diancourt et al. (2010)
*rpoB*	F: TCG TCG GCA GCG TCA GAT GTG TAT AAG AGA CAG GGC TTC TGA AGT ACG TGA CGT R: GTC TCG TGG GCT CGG AGA TGT GTA TAA GAG ACG TCA CGT GCA ACG TTC GCT T	502	Diancourt et al. (2010)

Citrate synthase (*gltA*); DNA gyrase subunit B (*gyrB*); glucose dehydrogenase B (*gdhB*); homologous recombination factor (*recA*); 60-kDa chaperonin (*cpn60*); glucose-6 phosphate isomerase (*gpi*); and RNA polymerase sigma factor (*rpoD*).

The genes were amplified from genomic DNA (1 μg), and PCR was performed under the following conditions: 94°C for 2 min, 35 cycles at 94°C for 30 s, 50°C for 30 s, and 72°C for 30 s, and a final step of 72°C for 5 min. To verify the amplified products *via* electrophoresis, 1.8% agarose gel electrophoresis was run using 1X TAE buffer at 120 V, and then the gel was stained with ethidium bromide to observe the amplified products with a transilluminator (Bio-Rad, CA, United States).

Constitutive gene sequencing was carried out using the Illumina Nextseq500 platform on 1 μg of genomic DNA. The readings obtained for each of the strains were analyzed through the bioinformatics tool FASTQC (Andrews, 2010) and were filtered with AfterQC (Chen et al., 2017). The sequences that had a depth of less than 20X and those that did not have the necessary length for the analysis were eliminated. Assembly of the readings was carried out from the sequences deposited in PubMLST using the aTRAM 2.0 program (Allen et al., 2018) with default parameters for paired readings and was verified by mapping the raw readings using BWA software (Li and Durbin, 2009). The mapped readings were filtered with SAMtools (Li et al., 2009) with the -m3 option to preserve only those readings that properly mapped onto the sequences, and the mappings were edited using SeaView (Gouy et al., 2010). The sequences obtained from each gene and the concatemer were analyzed using the database for *A. baumannii*.^1^ Each strain was characterized by a pattern of numbers that define its ST.

### CRISPR-Cas System Identification

The CRISPR-Cas I-Fb system (95°C for 5 min, 30 cycles at 95°C for 1 min, 58°C for 1 min, 72°C for 7 min, and a final step of 72°C for 10 min), the CRISPR AYE system (95°C for 5 min, 30 cycles at 95°C for 1 min, 45°C for 1 min, 72°C for 7 min, and a final step of 72°C for 10 min), and the gene *cas1* (95°C for 5 min, 35 cycles at 95°C for 30 s, 48°C for 30 s, 72°C for 30 s, and a final step of 72°C for 7 min) were identified by PCR (Table 3). To verify the presence of the amplified DNA, electrophoresis was performed on a 1% agarose gel using 1X TBE buffer at 120 V, and then the gel was stained with ethidium bromide to observe the amplification products with a transilluminator. The PCR products were purified and subsequently sequenced by capillary electrophoresis following the Sanger method. The sequences obtained were compared with the sequence of the CRISPR loci in strains of *A. baumannii*, which is available on the CRISPR web server.^2^ This server has 12 genomes of *A. baumannii* with structures confirmed by the CRISPR systems. The repeated sequences were analyzed by multiple alignments using the MultAlin interface page^3^ to determine the similarity between the strains.

**Table 3:** Primers used to amplify CRISPR-Cas systems.

Gene	Sequence 5'-3'	Reference
*aye-cris*	F: CCGTAGTTGAATCAACACGTA R: TTTGATTGGGTAAAATGCCAAA	Hauck et al. (2012)
*aye-cas1*	F: TCAAGCTGCGATGCGAATGT R: ATCCGGGCAAATTGAAACGC	Hauck et al. (2012)
*ab-cris*	F: AGTCCCAGAGTTTTGACCCA R: TTGGATTGGGTCATCATTGGT	Karah et al. (2015)

*aye-cris*: CRISPR-AYE system, *aye-cas1*: *cas1* gene, *ab-cris*: CRISPR-Cas I-Fb system.

### Statistical Analysis

The data were analyzed using the chi square test to evaluate the relationship between variables, with *p* < 0.05 considered significant. The descriptive statistics included percentages and frequencies.

## Results

### The MALDI-TOF-MS Biotyper Allowed the Differentiation of *Acb* Complex

The eighty-eight strains firstly identified as *A. baumannii* and 48 as *Acb* complex were reidentified with the MALDI-TOF-MS Biotyper, allowing us to differentiate between the species with the *Acb* complex. Additionally, 89.77% (79/88) of the strains were identified as *A. baumannii*, 6.82% (6/88) as *A. pittii*, and 3.40% (3/88) as *A. nosocomialis*.

### Patients in the Intensive Care Unit and Blood Culture Samples Were the Most Frequent Sources of *A. baumannii* Isolation

**Figure 1:**
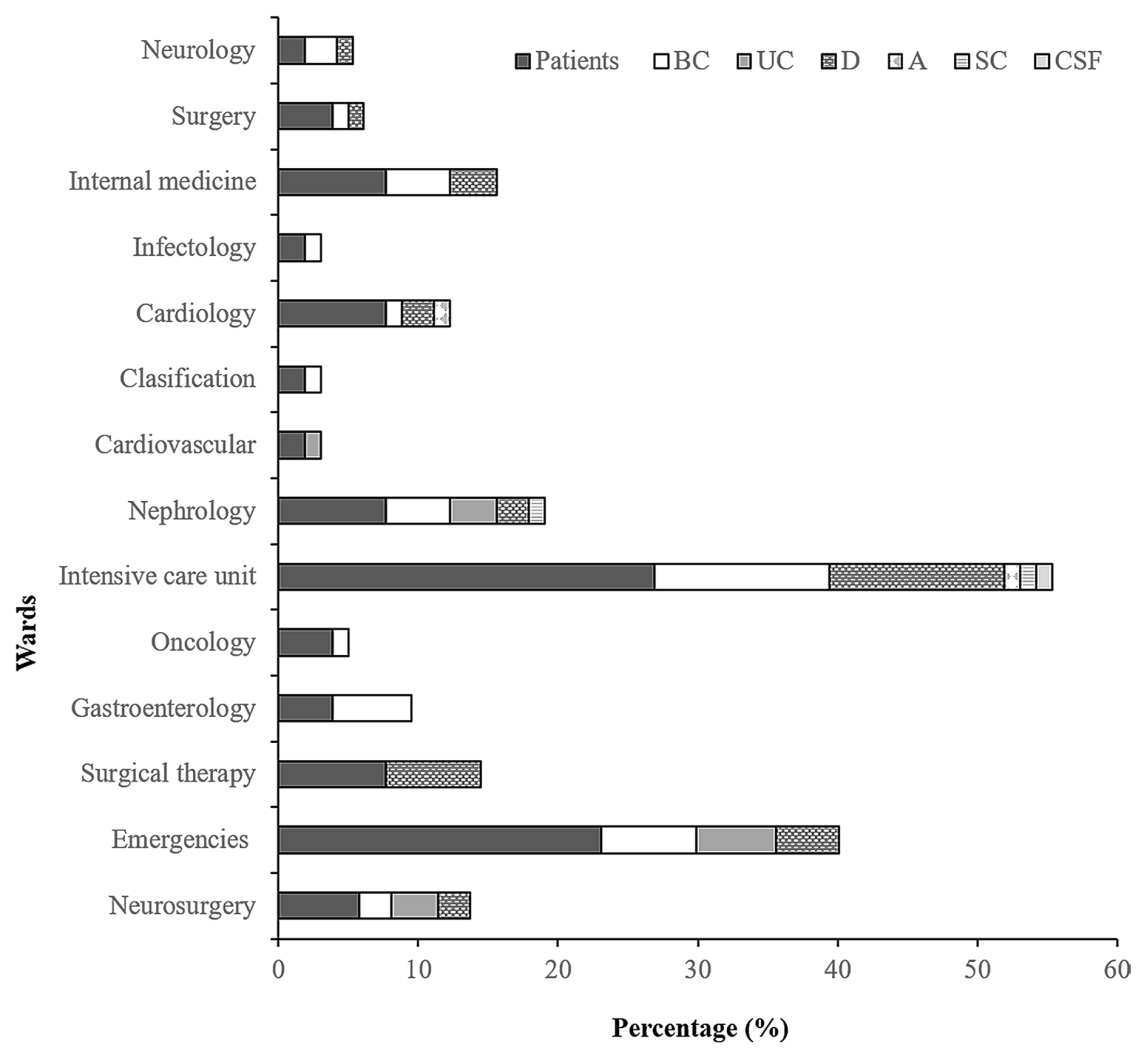
Frequency of isolation of the *Acinetobacter baumannii*, *Acinetobacter pittii*, and *Acinetobacter nosocomialis* strains. Percentage of patients from wards in which at least one of the strains was isolated in the period of study. BC, blood cultures; D, different samples; UC, urine cultures; SC, stool culture; A, autopsy; and CSF, cerebrospinal fluid.

In this study was included 52 patients (children between 1 and 15 years old) retained in 14 wards at the HIMFG from January 2015 to September 2017. Patients located in the ICU and emergency wards [26.92% (14/52)] made up the largest number of patients carrying the *Acinetobacter* strain (Figure 1).

From the 52 patients were recovered 88 strains with 37.5% (33/88), 25% (22/88), and 37.5% (33/88) obtained from the 2015, 2016, and 2017 samples, respectively. *A. baumannii* strains were identified in patients from several services wards, but mainly in emergency (13.63%, 12/88) and ICU (27.27%, 24/88). *A. pittii* strains were recovered from samples from patients in emergency, nephrology, and infectology. *A. nosocomialis* strains were obtained from samples from patients in surgery, gastroenterology, and internal medicine (Supplementary Table 1).

The isolation frequency by sample origin was as follows: 44.32% (39/88) from BC, 36.36% (32/88) from different (D) samples (including bronchial aspirate, catheter, mediastinal tissue, cholesteatoma culture, and peritoneal fluid), 13.63% (12/88) from urine cultures (UC), 2.27% (2/88) from a stool culture (SC) and an autopsy (A), and 1.14% (1/88) from cerebrospinal fluid (CSF, Figure 1).

According to the epidemiology analysis from 2015, nine strains (568BC, 173BC, 180BC, 181BC, 49BC, 50BC, 470BC, 471BC, and 800D) isolated from five patients were related to HAIs. Eight strains (219BC, 182BC, 183BC, 144D, 600BC, 928BC, 940BC, and 136BC) isolated from five patients were related to HAIs in 2017. The 13.63% (12/88) of strains were associated with primary bacteremia, 3.4% (3/88) with secondary bacteremia, and 2.3% (2/88) with pneumonia. The aforementioned infections were caused by identified strains such as *A. baumannii*, with the exception of one strain of *A. nosocomialis* (182BC) that was associated with primary bacteremia. Unlike these years in 2016, no HAIs were associated with *Acinetobacter* species.

### The *A. baumannii* and *A. pittii* Strains Were Multidrug Resistant

**Figure 2:**
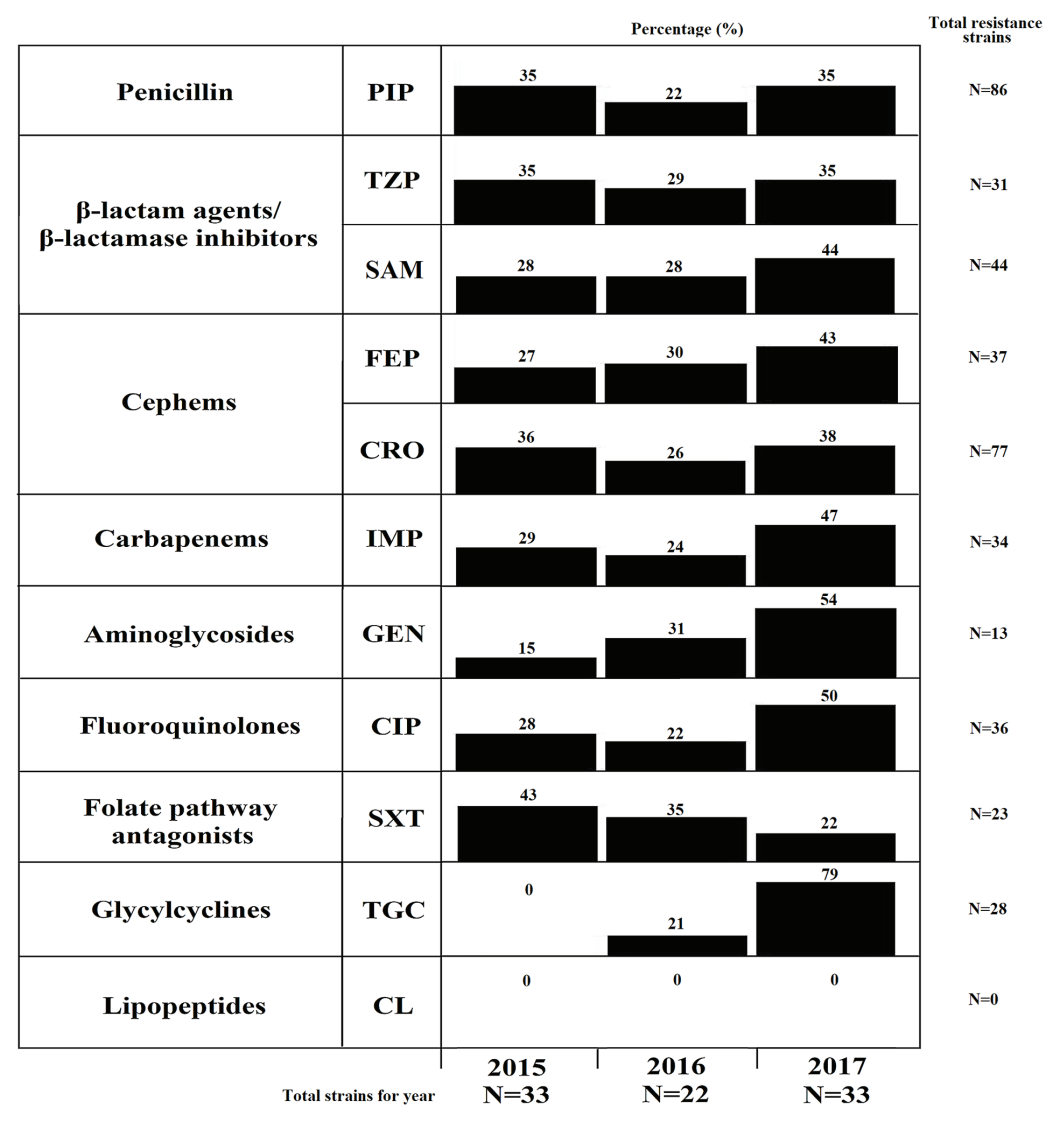
Resistance profiles of the *A. baumannii*, *A. pittii*, and *A. nosocomialis* strains. Percentages of strains resistant to at least one antibiotic in the following groups: penicillins (piperacillin), β-lactam agents/beta-lactamase inhibitors (ampicillin-sulbactam and piperacillin-tazobactam), third- and fourth-generation cephalosporins (cefepime and ceftriaxone), carbapenems (imipenem), aminoglycosides (gentamicin), fluoroquinolones (ciprofloxacin), folates (trimethoprim/sulfamethoxazole), glycylcyclines (tigecycline), and lipopeptides (colistin). PIP, piperacillin; TZP, piperacillin/tazobactam; SAM, ampicillin-sulbactam; FEP, cefepime; CRO, ceftriaxone; IMP, imipenem; GEN, gentamicin; CIP, ciprofloxacin; SXT, trimethoprim/sulfamethoxazole; TGC, tigecycline; and CL, colistin.

The resistance profile was as follows: 97.73% (86/88) to a penicillin (piperacillin), 50% (44/88) to β-lactam combination agents (ampicillin-sulbactam), 35.23% (31/88) to β-lactam combination agents (piperacillin-tazobactam), 87.5% (77/88) to a third-generation cephem (ceftriaxone), 42.05% (37/88) to a fourth-generation cephem (cefepime), 38.64% (34/88) to a carbapenem (imipenem), 14.77% (13/88) to aminoglycosides (gentamicin), 40.91% (36/88) to fluoroquinolone (ciprofloxacin), 26.14% (23/88) to folate pathway antagonists (SXT), and 31.82% (28/88) to a glycylcycline (tigecycline; Figure 2). All strains were susceptible to colistin.

**Figure 3:**
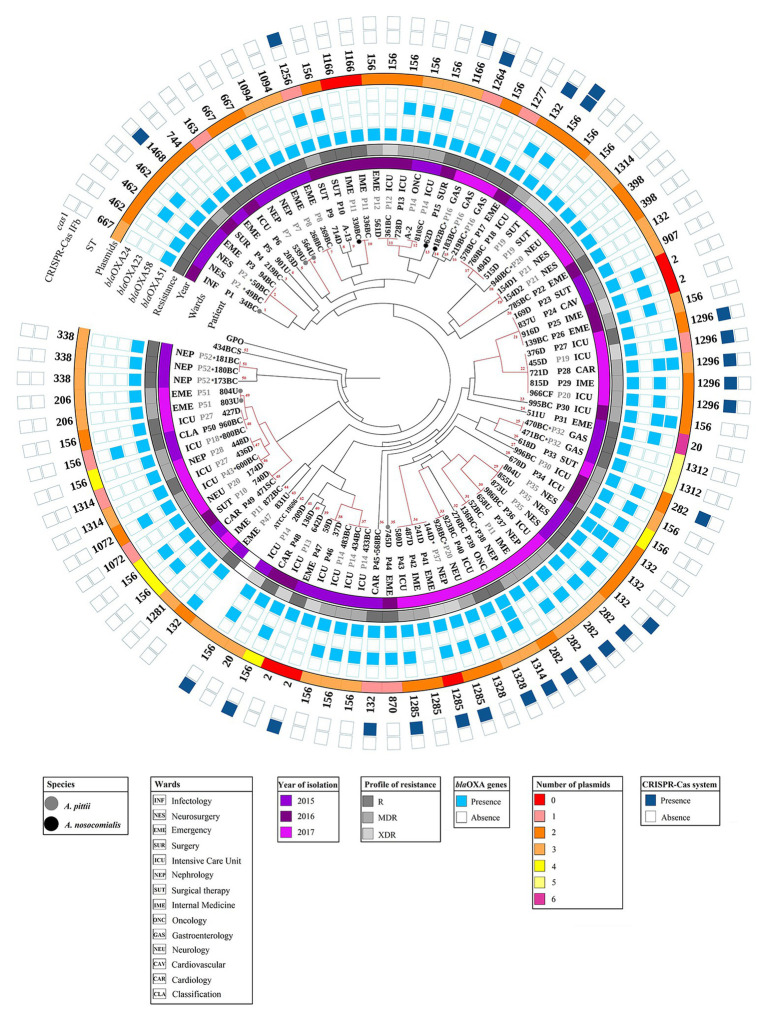
Dendrogram profile of the *A. baumannii*, *A. pittii*, and *A. nosocomialis* complex strains. Dendrogram profile displaying the genetic relatedness among the 88 strains based on the pulsed-field gel electrophoresis (PFGE) pattern. The red line represents the strains with the same pulsotype. The species other than *A. baumannii* are shown with circles in gray (*A. pittii*) and black (*A. nosocomialis*). Internal data represent strain with the origin of isolation, followed by the patient and the ward of origin for each strain. The asterisk symbol shows the strains associated with HAIs (bacteremia and pneumonia). The first band represents the year of isolation, and the second represents the profile of resistance: Resistant (R), multidrug-resistant (MDR), and extensively drug-resistant (XDR). The strains with a R profile showed resistance to PIP, SAM, or TZP. The detection of *bla_oxa_* genes is represented by clear blue clear squares and the amplification of the CRISPR-Cas system with dark blue squares. The number of plasmids associated with each strain is shown in the third band. The type sequences (ST) for each strain were added. The color codes for each analyzed characteristic are shown in the legends below the figure. The visualization was performed with the iTol program (Letunic and Bork, 2007).

Additionally, 44.32% (39/88) of the strains showed a MDR resistance profile. The MDR profile distribution according to species was as follows: 94.87% (37/39) for *A. baumannii* and 5.13% (2/39) for *A. pittii* strains (Figure 3; Supplementary Table 1). Interestingly, the number of resistant strains for imipenem and tigecycline increased (*p* < 0.05) during 2017 (Supplementary Table 2). This is in contrast to folate pathway antagonists, for decreases in the number of resistant strains were observed (*p* < 0.05). On the other hand, 11.36% (10/88) of strains showed an XDR profile. Interestingly, all strains with this profile were identified as *A. baumannii* (Figure 3; Supplementary Table 1).

### The *bla*_OXA-23_ Gene was Amplified in the *A. baumannii* and *A. pittii* Strains

The presence of *bla*_OXA-LIKE_ genes related to carbapenem resistance was determined by PCR. All strains amplified the *bla*_OXA-51_ gene, 51.13% (45/88) the *bla*_OXA-23_ gene, 4.54% (4/88) the *bla*_OXA-24_ gene, and 2.27% (2/88) the *bla*_OXA-58_ gene (Figure 3). Interestingly, the *bla*_OXA-23_ gene was amplified in two species (*A. pittii* and *A. baumannii*). The *bla*_OXA-24_ and *bla*_OXA-58_ genes were amplified only *A. baumannii* strains (Figure 3; Supplementary Table 1).

### One to Six Plasmids Were Identified in *A. baumannii*, *A. pittii*, and *A. nosocomialis* Strains

The plasmid profiles obtained through the Eckardt method showed that the strains had from one to six plasmids with sizes between 2.4 and 121 kb. The results were the following: 37.5% (33/88) of the strains presented two plasmids, 34.09% (30/88) presented three plasmids, and only one of the strains presented six plasmids; however, in 7.95% (7/88) of the strains, these mobile elements were not detected (Figure 3; Supplementary Table 1). Briefly, *A. baumannii* strains carried one to six plasmids, *A. pittii* strains carried two or three plasmids, and *A. nosocomialis* carried one or three plasmids. No correlation was found between the resistance profile and the plasmid number; however, the largest number of MDR strains harbored between two or three plasmids.

### The *A. baumannii* Strains Were Genetically Diverse in Comparison to *A. pittii*, *A. nosocomialis*

Macrorestriction patterns obtained by PFGE showed between 16 and 23 fragments with sizes of 48–339 kb. For construction of a dendrogram, a sensitive strain (434S), and the ATCC®19606 strain were included. In agreement with the macrorestriction pattern, the strains were distributed in 52 pulsotypes, and 24 showed were closely related with a correlation coefficient of 1.0 (Figure 3). Most of the strains maintained a correlation between the number of plasmids obtained by the Eckard technique and its pulsotype.

Strains recovered from the same patient were grouped into pulsotypes 6, 7, 10, 12, 19, 25, 37, 49, and 51 (Figure 3). The isolation period of these strains in the same patient was a maximum of 5 days according to PFGE and other determinants analyzed (resistance profile, *bla_oxa-like_* genes, plasmid number, ST, and CRISPR-Cas system) in this study, suggesting the presence of the same strain.

Interestingly, 12 pulsotypes (2, 11, 17, 21, 22, 31, 33, 34, 38, 46, 47, and 48) harbored strains with an identical macrorestriction pattern, which were isolated from at least two different patients with a minimum difference of 1 month between each strain. However, some changes were observed between these strains, mainly in profile resistance and *bla_oxa-like_* gene amplification.

Notably, some of the strains grouped in the same pulsotypes were isolated from different patients and/or wards and in some cases from samples from different years, such as, pulsotypes 17, 21, 31, and 45, suggesting the persistence of more than one clone in the hospital environment (Figure 3).

*A. pittii* strains were clustered into pulsotypes 1, 6, 35, and 49; while *A. nosocomialis* strains were clustered into pulsotypes 10, 13, and 14. Interestingly, all *A. nosocomialis* strains were closely related to *A. baumannii* strains. In addition, pulsotypes 2, 14, 15, 16, 18, 25, 31, 33, 34, 36, 46, 48, 50, and 51 harbored the strains related with HAIs (Figure 3; Supplementary Table 1).

### ST 156 was Widely Distributed in the *A. baumannii* Strains

MLST results from *Acinetobacter* strains showed 27 STs, of which the most prevalent was ST 156, at 27.27% (24/88), followed by ST 132, 7.95% (7/88); STs 1296 and 282, 5.68% (5/88); STs 2, 1285, and 1314, 4.54% (4/88); STs 338, 462, 667, and 1166, 3.41% (3/88); STs 20, 206, 398, 1072, 1094, 1312, and 1328, 2.27% (2/88); and STs 163, 744, 870, 907, 1256, 1264, 1277, 1281, and 1468, 1.14% (1/88).

The relationships between the STs obtained in this study were analyzed through eBURST, which found that they belong to 12 clonal complexes (CC20, CC64, CC79, CC132, CC163, CC214, CC462, CC629, CC782, CC1094, CC1264, and CC2), and the last clonal complex (CC2) harbored most of the STs (Figure 3; Supplementary Table 1). The results obtained with MLST remained correlated with the data generated data through the other typing tools used in this study. The strains that presented the same plasmid, pulsotype, and resistance profiles also showed the same STs. Two inconsistencies were found between the PFGE and MLST results; more than one ST in the strains grouped into 17 and 21 pulsotypes, suggesting the presence of different strains in the same pulsotype.

In relation to the *A. pittii* strains, three STs (206, 667, and 870) were identified and regarding the *A. nosocomialis* strains, two STs (1166 and 1264) were identified (Figure 3; Supplementary Table 1). Interestingly, ST 1166 identified in *A. nosocomialis* (strains 330BC and 62D) has been described for *A. baumannii*, and according to the pulsotypes, these strains showed a macrorestriction pattern related to *A. baumannii* strains (pulsotypes 10 and 13). *A. nosocomialis* ST 1264 was associated with CC1264, while in *A. pittii*, only ST 667 was found in CC21; the other ST has not been associated with a CC.

Strains associated with HAIs showed STs 132, 462, 1312, 156, 338, 282, 1264, 1277, 1285, 1072, 1314, and 1328 (Figure 3; Supplementary Table 1)^4^.

### CRISPR-Cas I-Fb Systems Were Identified in the *A. baumannii*, and *A. nosocomialis* Strains

The CRISPR-Cas I-Fb system was amplified in 22.73% (20/88) of the *A. baumannii* and *A. nosocomialis* strains; however, the other CRISPR-AYE system was not amplified (Figure 3; Supplementary Table 1). The *cas1* gene was identified in 7.95% (7/88) of the strains.

The CRISPR-Cas I-Fb system was identified based on the sizes of the amplified PCR products, which was determined through sequencing spacers and repeated sequences. The sizes of the amplified sequences were approximately 700 bp (data not shown). Thereafter, they were sequenced, and when the bioinformatics search was carried out on the CRISPR server, it was found that the CRISPR-Cas I-Fb system sequences had 10 spacers with identical sequences to the nucleotide sequences of the strains in which they were performed^5^.

## Discussion

*A. baumannii* is an opportunistic pathogen that is associated with severe infections worldwide and is related to the different attributes that allow it to emerge as a nosocomial microorganism, i.e., mainly resistance to antibiotics and its ability to persist in hospital environments due to its ability to resist drying (Antunes et al., 2014; Harding et al., 2017).

The genus *Acinetobacter* have six species with very similar phenotypes they and have been grouped into the *Acb* complex. Of this complex, *A. pittii*, *A. nosocomialis* and *A. baumannii* are the species associated with a greater number of infections and mortality; in addition, the isolation frequency of *A. pittii* and *A. nosocomialis* strains as etiological agents of HAIs is high (Wisplinghoff et al., 2012). The mortality rates of *A. pittii* are greater than those of *A. nosocomialis* but lower than those of *A. baumannii*, suggesting that a future *A. pittii* strain could also emerge as an important nosocomial pathogen (Yang et al., 2012). For this reason, the molecular typing of the *Acb* complex using different techniques is essential to generate information about the epidemiology of this complex and to learn more about the distribution of these species in hospital environments. The differentiation of *Acb* complex strains was carried out through MALDI-TOF MS in this study. This method allowed for the identification of the *A. baumannii* strains and differentiation between the species that belong to the *Acb* complex (Lin et al., 2008); three species were identified in this study: *A. baumannii*, *A. pittii*, and *A. nosocomialis*.

The ward with the highest recurrence of *A. baumannii* was the ICU, which corresponded to the data from other regions such as Europe, Asia, the United States, Latin America, and Morocco (Martins et al., 2009; Vincent et al., 2009; Uwingabiye et al., 2017). The high recurrence of isolating *A. baumannii* in the ICU has been attributed to risk factors for the development of infections, including invasive procedures, catheter placement, and intubation endotracheal, which may cause urinary tract and respiratory tract infections (Cisneros et al., 1996; Lynch et al., 2017). However, the development of *A. baumannii* infections is also related to the immune systems of patients since it has been observed in individuals with serious diseases such as hematological malignancies and diabetes mellitus, and even those subjected to prolonged antimicrobial therapy with broad-spectrum antibiotics (Vincent et al., 2009; Kempf and Rolain, 2012; Lin et al., 2016). *A. pittii* and *A. nosocomialis* strains were identified in patients at the HIMFG, but none of these strains were isolated from the ICU ward. The resistance percentage found in this study was low compared with those in other studies (Alcántar-Curiel et al., 2014, 2019; Xie et al., 2018).

The low resistance percentages could be associated with the source of strains from pediatric patients; however, an increase in the number of imipenem-resistant strains was found in 2017. The frequency of MDR strains in this study was high (44.32%), and this phenotype was identified in *A. baumannii*, and *A. pittii* strains. This MDR profile contrasted with the XDR profile, as only *A. baumannii* strains had this phenotype, and resistance has been associated with high epidemic potential and high mortality (Song et al., 2011; Lee et al., 2014). The main mechanism of *A. baumannii* resistance to β-lactams is enzymatic degradation by β-lactamases; oxacillinases (OXAs) give it the ability to hydrolyze oxacillin (Evans and Amyes, 2014). In this study, the *bla*_OXA-51_ gene, in addition to the *bla*_OXA-23_, *bla*_OXA-24_ and *bla*_OXA-58_ genes (identified more frequently in *A. baumannii*), were identified by PCR. All of the strains amplified the *bla*_OXA-51_ gene, which has been reported to be intrinsic in *A. baumannii*, with *bla*_OXA-69_ (Brown et al., 2005). The chromosomal location and the detection of these genes allow for identification at the species level (Turton et al., 2006).

Strains with an amplified *bla*_OXA-23_ gene formed the first group identified in *A. baumannii*, and the production of this enzyme is sufficient to confer resistance to carbapenems. This group is mostly distributed and has been detected in clinical strains in Brazil, Belgium, Singapore, and France, where have been related to outbreaks. In addition, the presence of the *bla*_OXA-23_ gene is limited to not only clinical strains but also environmental isolates, suggesting that the propagation of these genes occurs in different environments and under different selective pressures (Girlich et al., 2010). Moreover, 27% of the strains (*A. pittii* and *A. baumannii*) that amplified the *bla*_OXA-23_ gene showed resistance to imipenem, which have been reported in Latin America and Mexico. Other determinants of resistance have been associated with these species, such as the *bla*_OXA-58_ gene and metallo-β-lactamases, which are predominantly responsible for carbapenems resistance in *A. nosocomialis* and *A. pittii*; however, *bla*_OXA-23_ and *bla*_OXA-24_ have recently become more common in carbapenems resistance for both species (Yang et al., 2012; Cayô et al., 2014; Silva et al., 2018).

Most of the elements that encode for resistance can be found in mobile elements, such as plasmids. The determination of plasmid profiles has been used for comparisons of strains, since these can be found in identical numbers, sizes, and molecular weights in bacteria with different isolation origins. Our results agreed with other studies in which the authors included the characterization of 132 clinical strains of *A. baumannii* harboring of 1 to 7 plasmids (Singh et al., 2006). Furthermore, the strains that presented the same pulsotype, and ST had identical numbers of plasmids and molecular weights, with the exception of pulsotypes 17, 22, and 34, which showed the same type sequence and pulsotype but different in resistance profiles, *bla*_OXA-LIKE_ genes, and plasmid profiles. The plasmid profile has been used as a complementary typing method to others such as PFGE; in this study, we observed congruence between the results obtained through each one; however, one of the limitations of this method is the use of mobile elements to carry out the typing of strains. Nevertheless, it provides a general overview of whether the strains can be carriers of mobile elements, from which their roles can be determined as mechanisms underlying the transfer of virulence and resistance determinants based on their sequencing.

Due to its high reproducibility, one of the most commonly used tools in the typing of clinical strains is PFGE. According to Tenover et al. (1995), when isolates do not differ in band pattern, they are considered indistinguishable, and therefore, the strains can be related. We found that 24 pulsotypes presented identical macrorestriction patterns; some strains were recovered from the same patient, but the others were isolated from different patients. The results of PFGE were consistent with the plasmid profiles, but when the advantages and disadvantages of these methods were compared, PFGE is clearly a technique that can be time consuming, laborious and expensive with respect to the profiling of plasmids; however, the results by PFGE were more accurate in terms of the typing of the strains, its reproducibility is high, and we can also obtain information regarding genetic diversity and clonal relationships.

Another one of the most common tools for typing *A. baumannii* strains is MLST, which is based on sequencing the variable regions of seven genes. For *A. baumannii*, two protocols based on different constitutive genes have been proposed, with some discrepancies (Tomaschek et al., 2016; Gaiarsa et al., 2019). Among the disadvantages that have been reported when using this method is the consideration of only seven constitutive genes of the total chromosomal information of *A. baumannii* strains, a bacterium with high genetic variation and would be disadvantageous to consider only a portion of the total information (Wang et al., 2013; Feng et al., 2016; Castillo-Ramírez and Graña-Miraglia, 2019). In this study, the strains were analyzed using the modified Pasteur protocol because in the case of the Oxford scheme, complications have been reported in the amplification of genes such as *gdhB* and *gpi* (Hamouda et al., 2010; Hamidian et al., 2017; Gaiarsa et al., 2019). The nucleotide sequences for the determination of STs were obtained through massive sequencing using next-generation platforms. However, under the Pasteur protocol, it was difficult to amplify and sequence the *fusA* gene; therefore, we designed other primers that allowed us to sequence this gene.

The most prevalent STs were STs 156, 132, 1296, 2, 282, 1285, and 1314. Interestingly, ST 156 belongs to the CC79 complex, which has been associated with outbreaks in several countries (Mexico, Canada, Honduras, Colombia, and even in Europe) and is epidemiologically important in Mexico (Loewen et al., 2014; Kubo et al., 2015; López-Leal et al., 2019). ST 156 strains are producers of carbapenemases (*bla*_OXA-23_) and recently included was the new variant OXA-239 (Tamayo-Legorreta et al., 2014; Graña-Miraglia et al., 2017; Mancilla-Rojano et al., 2019). According to our results, there is an association between ST 156 and *bla*_OXA-23_ gene amplification and between MDR and XDR profiles. Interestingly, ST 156 was identified during all 3 years of this study, which indicates that this lineage has been maintained and distributed within the hospital. However, other STs were associated with strains of a specific year. These data suggest that there is more than one strain distributed simultaneously within the HIMFG. STs 132 and particularly ST 2 the most common clone globally, are distributed in Europe, Asia, and Latin America, associated with outbreaks and resistance to carbapenems due to the presence of the *bla*_OXA-66_ and *bla*_OXA-120_ genes (Bakour et al., 2014; Dahdouh et al., 2017; Nowak et al., 2017; Levy-Blitchtein et al., 2018; Villalón et al., 2019). Conversely, we report a strain associated with ST 1468 that has recently been entered into the MLST *A. baumannii* database.

This technique has been used to distinguish the species of the *Acb* complex and designed to study the population of various bacteria, showing the presence of different clonal lineages, which could, for example, be associated with different species of the *Acb* complex (Bartual et al., 2005; Yamada et al., 2016). This finding agrees with our results, since the STs obtained for *A. pittii* were described in the MLST database for this species. Interestingly, the STs identified for *A. pittii* and *A. nosocomialis* are associated with different clonal complexes from those associated with *A. baumannii*. The MLST protocol is expensive and time consuming, and the analysis of the data can be slow; however, it should be noted that it allows the typing of strains and the differentiation of some species of the *Acb* complex (such as *A. pittii*). Similarly, the comparison with strains in other parts of the world enabled us to recognize the STs and clonal complexes associated with this hospital, determining that ST 156 is a clone that persists in the HIMFG.

The PFGE results were consistent with the MLST results, the strains with the same pulsotype presented the same ST, and except for the strains with pulsotypes 17 and 21, which were related despite being associated with different patients and years. The pulsotypes 17 and 21were grouped with the same restriction patterns; nevertheless, we could observe that they differed with respect to their resistance profile, plasmid profile, and amplification of *bla_OXA_* genes. However, the determination of different STs allows us to suggest that these pulsotypes group different strains; the opposite occurred in some of the strains that were isolated from different patients, since there were cases in which, according to the agreement of the data obtained by PFGE, the same patient could harbor different pulsotypes; nevertheless, they could present the same ST (P14, and P30).

Recently, the polymorphism observed between CRISPR-Cas systems has been used to genotype strains and establish phylogenetic relationships in different bacterial species; although, in some cases the spacers can be too diverse, hindering their use as a method for subtyping. On the other hand, the CRISPR-Cas system has been implemented as a successful typing method in species such as *Salmonella enterica* and *Yersinia pestis* (Cui et al., 2008; Shariat et al., 2015). The use of this system has also separated other bacteria such as *Erwinia amylovora* into different groups depending on their geographical origin (Rezzonico et al., 2011). Two systems have been described in *A. baumannii*: CRISPR-AYE and CRISPR-Cas subtype I-Fb (Hauck et al., 2012; Karah et al., 2015). In this study, the CRISPR-Cas subtype I-Fb system was identified in only 20 strains. Despite the amplification of the CRISPR-AYE system with primers reported previously by Karah et al. (2015), when the PCR products were sequenced, the nucleotide analyses did not show any homology with the CRISPR-Cas systems.

Despite the successful implementation of the CRISPR-Cas system as a typing method in other studies, we were unable to use it because not all of the strains had this system; however, two phenomena were observed. The first was that some strains that amplified the CRISPR-Cas I-Fb system had been grouped in the same pulsotypes and possessed the same STs. The second was that some strains had the same pulsotype but differed in STs or CRISPR-Cas system; interestingly, they provide us with information on the diversity within each of the subgroups obtained by PFGE and MLST. There was no ST that was specifically associated with some type of these systems, but interestingly, the CRISPR-Cas I-Fb system was identified with more frequency in strains recovered during 2017.

On the other hand, approximately 70% (14/20) of the strains with a CRISPR-Cas I-Fb system presented MDR and XDR profiles, which has not been observed in other studies since there is no correlation between the previous profiles (Hauck et al., 2012). Amazingly, we found that the strains that amplify the CRISPR-Cas I-Fb system presented from 1 to 4 plasmids. According to Mangas et al. (2019), there is an association between the presence of the CRISPR-Cas system and the absence of plasmids in *A. baumannii* genomes; as we could see in our results; these systems would limit the transfer of mobile elements such as plasmids.

In addition, the gene encoding the Cas1 protein of the CRISPR-Cas system was also identified only in seven strains. The gene encoding the Cas1 protein is one of the most conserved and is involved in the acquisition of spacers and it has been proposed that the gene evolves slower than other *cas* genes (Takeuchi et al., 2012; Makarova and Koonin, 2015). The strains that amplified this gene did not amplify any CRISPR-Cas system, so we could suggest the presence of another system different from those sought in this study. In contrast, 26 strains amplified at least one system but not the *cas* gene. For *Enterococcus faecium* strains, the *cas1* gene has not been identified with CRISPR-Cas IIA systems, suggesting that these systems have lost their ability to acquire different spacers (van Schaik and Willems, 2010; Lyons et al., 2015), which could also have occurred in our strains. However, it is necessary to carry out other studies to demonstrate this hypothesis.

The amplified spacers were larger in the CRISPR-Cas I-Fb system. Strains that had more spacers in their CRISPR systems have been related to evolution in the environment (Hauck et al., 2012). The detection of these systems through PCR and amplification sequencing seems to be simple; however, something that must be highlighted is the probable implication of these systems in other processes. CRISPR-Cas systems in *E. coli* are related to the repair of DNA damage (Babu et al., 2011); this would be relevant in *A. baumannii*, since microorganisms are subjected to a series of environmental agents or possess resistance to desiccation. An important contribution of our work is the identification of the CRISPR-Cas system in clinical strains.

In the case of patients, there were two interesting conditions. First, in patients (14, 20, 27, 28, 37, 43, and 47), more than one strain was isolated, some with differences of days or 1 month. These strains were all different, locating themselves into different pulsotypes, STs, and with differences in the rest of the characteristics evaluated, suggesting the presence of different strains in the same patient over a short temporality. In the second case, was observed that patients (11, 16, and 19) had more than one strain; however, they were divided into two groups in the same patient that were identical, and that were not same in the characteristics evaluated, suggesting once again the presence of more than one strain over a longer period. In 11 patients, the strains were obtained on the same date but corresponded to different samples, presenting cases, in which the strains showed the same characteristics with respect to the pulsotype, *bla*_OXA-LIKE_ genes, plasmid profile, and type sequence. Nevertheless, five patients (P16, P18, P27, P30, and P47) provided samples on the same date but with phenotypic and genotypic characteristics that were completely different. Also, there were cases presented in which the samples obtained more than 1 day apart exhibited the same characteristics.

Only 17 strains were associated with HAIs, such as bacteremia and pneumonia. According to the results, the patients: 2 (49BC and 50BC), 32 (470BC and 471BC), and 52 (173BC, 180BC, and 181BC) related with bacteremia, had more than one strain. The strains were distributed along the tree and only clustered when they were from the same patient such as pulsotypes 2 and 25, for which all of the phenotypic and genotypic characteristics were identical, with the exception of strain 173BC recovered from the patient 52, the only difference that had with the other two strains (189BC and 181BC) isolated of this patient was his macrorestriction pattern. Patients 18 (800BC) and 38 (136BC) developed bacteremia; while, patients 37 (144D), 43 (600BC), and 45 (568BC) developed pneumonia, the strains were randomly distributed in different pulsotypes, but apparently there was no related between them. Finally, patients 16 (182BC, 183BC, and 219BC) and 20 (928BC and 940BC) with bacteremia were all different from each other. Interestingly, these features were observed for strains identified as *A. baumannii*, with the exception of one strain of *A. nosocomialis* that was associated with a primary bacteremia. The latter was isolated together with two strains of *A. baumannii* from the same patient (P16) on the same date; however, they presented different genetic characteristics, which suggest that more than one different strain could be causing an infection in the patient. These strains were collected from a total of 10 patients and are genetically diverse since they presented different plasmid profiles, type sequences, and pulsotypes.

According to the results obtained in this study, an increase was identified an increase in the number of resistant carbapenems strains. The molecular typing methods allowed us to determine the relationships between clinical strains. PFGE data demonstrated that different patients could be infected by the same strain (identical pulsotypes were harbored by more than one patient). MLST showed that the strains in one group, i.e., pulsotypes 17 and 21, showed different STs and thus were not the same strain. ST 156 is a persistent clone at HIMFG. Finally, the CRISPR-Cas system identified in a low percentage of these strains could be associated with the nosocomial environment. The severity of the disease and the difference between strains that are closely related to those associated with HAIs shown in this study, were related to the immunocompromise patient and virulence factors of the strain, which were not determined in this study and will be examined in a future study. The clinical importance of *A. baumannii* in ICUs isolated from BCs has been well documented. *A. baumannii* infections (i.e., nosocomial pneumonia) tend to be more serious than those caused by *A. nosocomialis* and *A. pittii* (Lee et al., 2013). Therefore, it is necessary to identify and differentiate the species in the *Acb* complex to determine their epidemiological and clinical importance. The implementation of molecular epidemiology allows the characterization of a set of strains and identification of different attributes associated with their distribution in a specific environment. This study will allow future interventions for the recognition of risk factors related to opportunistic pathogens such as *A. baumannii*.

## Data Availability Statement

The datasets presented in this study can be found in online repositories. The names of the repository/repositories and accession number(s) can be found in the article/Supplementary Material.

## Ethics Statement

The Research Committee (Dr. Juan Garduño Espinosa), Ethics Committee (Dr. Luis Jasso Gutiérrez), and Biosecurity Committee (Dr. Marcela Salazar García) of the HIMFG granted approval for the development of the protocol HIM/2017/003 SSA.1299, HIM/2018/038 SSA.1513. The strains were provided by the Central Laboratory of the HIMFG, with prior informed consent of the patients to obtain the samples. Written informed consent was not required for this study according to the institutional ethical, biosecurity and investigation committees because the Central Laboratory from the HIMFG provided the *A. baumannii* clinical strain isolates from the child included in this study.

## Author Contributions

AC-C had the initial idea and developed it into a project together with JX-C. JM-R done the experiments. AC-C, JX-C, SO, VF, and JA-G analyzed the data. OM-C performed identification by MALDI-TOF Biotyper. DR-Z reviewed the clinical data. AC-C, JX-C, SO, KE-M, MC, JR-G, JA-G, RH-C, and OM-C contributed reagents and materials. IP-O supplied the *A. baumannii* strains. MC-C carried out susceptibility. AC-C and JX-C wrote the manuscript, read and approved the final version. All authors contributed to the article and approved the submitted version.

### Conflict of Interest

The authors declare that the research was conducted in the absence of any commercial or financial relationships that could be construed as a potential conflict of interest.

## Footnotes

^1^http://pubmlst.org/abaumannii/

^2^https://crispr.i2bc.paris-saclay.fr/

^3^http://multalin.toulouse.inra.fr/multalin/

^4^https://github.com/JetsiMancilla/MLST-A.baumannii

^5^https://github.com/JetsiMancilla/CRISPR-Cas-System
